# Physics-constrained GAN boosts OAM correction in ocean turbulence

**DOI:** 10.3389/frai.2025.1702056

**Published:** 2026-01-12

**Authors:** Xiaoji Li, Zhiyuan Wang

**Affiliations:** Key Laboratory of Cognitive Radio and Information Processing, Ministry of Education, Guilin University of Electronic Technology, Guilin, China

**Keywords:** machine learning, physics-constrained GAN, OAM, oceanic turbulence correction, underwater optical communication

## Abstract

**Introduction:**

This study addresses the challenge of improving wavefront correction for Orbital Angular Momentum (OAM) in oceanic turbulence using a physics-constrained Generative Adversarial Network (GAN).

**Methods:**

We integrated physical constraints into a deep learning framework to reconstruct degraded input images (SSIM = 0.62). The model was trained with varied loss settings, including a baseline model, spectral constraints (+Spec), and spatial constraints (+Ortho).

**Results:**

The dual-constraint approach (+Ortho+Spec) reached a near-optimal SSIM of 0.98. Ablation studies revealed that while +Ortho boosted modal purity to 95.7%, the dual-constraints achieved 98.4% purity. Power spectral density analysis via KL divergence confirmed the dual-constraints' superiority (KL = 0.56) over the baseline (KL = 2.47).

**Discussion:**

These results demonstrate that integrating both spatial and spectral constraints effectively optimizes reconstruction, purity, and spectral fidelity, offering a robust solution for OAM correction in underwater optical communication systems.

## 1 Introduction

Underwater optical wireless communication (UOWC) systems have emerged as a promising technology for high-bandwidth data transmission, yet they face fundamental limitations due to the complex nature of the oceanic channel (Vali et al., 2025; Baykal et al., 2022). Among the various multiplexing techniques, Orbital Angular Momentum (OAM) beams are particularly valuable for increasing channel capacity, as their helical phase fronts carry distinct topological charges that serve as independent information carriers (Wang and Willner, 2014; Guo et al., 2023). However, the reliability of OAM-based links is severely compromised by oceanic turbulence, which induces complex inhomogeneities in the propagation medium.

It is crucial to distinguish the specific impact of wavefront distortions from general intensity fluctuations in OAM systems. While oceanic turbulence manifests as both scintillation (intensity fluctuation) and phase aberrations, their effects on signal integrity differ fundamentally. Intensity fluctuations primarily degrade the Signal-to-Noise Ratio (SNR), leading to signal fading. In contrast, wavefront distortions directly perturb the helical phase structure that defines OAM modes. This phase disruption destroys the orthogonality between modes, inducing severe inter-modal cross-talk that cannot be resolved by simple intensity smoothing. Therefore, restoring the wavefront's structural integrity—specifically the phase singularity—is a prerequisite for recovering the channel capacity, necessitating correction strategies that go beyond mere image denoising (Subramaniam et al., 2020).

In response to these challenges, the integration of Machine Learning (ML) and Deep Learning (DL) into optical communication systems has evolved significantly in recent years. A comprehensive survey by Amirabadi et al. highlights the growing reliance on these data-driven techniques for channel modeling and signal processing (Amirabadi et al., 2024). In the realm of UOWC, Deep Reinforcement Learning (DRL) has emerged as a powerful tool for system optimization. For instance, Rathour utilized DRL to enhance link performance in dynamic underwater environments (Rathour, 2023), while Shin et al. applied similar strategies for multidimensional beam optimization (Shin et al., 2024). Complementing these adaptive approaches, Simon et al. proposed energy-adaptive neural networks to improve efficiency in underwater IoT networks (Simon et al., 2025), and Shafi et al. explored spatial diversity techniques to mitigate oceanic channel impairments (Shafi et al., 2025). Beyond system-level optimization, DL architectures have been successfully deployed to improve signal integrity, with Iqbal et al. demonstrating significant reductions in symbol error rates for short-reach optical networks (Iqbal et al., 2024). Regarding OAM systems specifically, Ye et al. developed hybrid optical-electronic Convolutional Neural Networks (CNNs) for efficient OAM demultiplexing (Ye et al., 2024), and Cai et al. achieved accurate recognition of composite vortex beams even under moderate-to-strong turbulence (Cai et al., 2024). To address turbulence-induced distortions, recent studies have advanced from general underwater image enhancement (Liu, 2025) to precise wavefront reconstruction. Baharlou et al. introduced hybrid networks for single-shot aberration correction (Baharlou et al., 2025), while Zhang et al. utilized neural operators for depth-heterogeneous turbulence correction (Zhang et al., 2025). Notably, Long et al. demonstrated that physics-informed neural networks could achieve in situ wavefront correction, validating the efficacy of combining data-driven learning with physical laws (Long et al., 2024).

Despite these advancements, generative models, particularly Generative Adversarial Networks (GANs), which treat wavefront correction as an image-to-image translation task, often show potential but face limitations (Pradhyumna and Mohana, 2022). Models like pix2pix map distorted intensity profiles to their ideal counterparts (Isola et al., 2016). However, these conventional implementations frequently overlook the inherent physical laws governing light-turbulence interactions (Newman et al., 2011). By relying predominantly on pixel-wise loss functions (e.g., L1 or L2 norms), standard GANs tend to optimize visual similarity (intensity restoration) while neglecting the preservation of topological charge integrity and spectral dynamics (Zhan et al., 2021).

This oversight results in two critical deficiencies in current methods. First, the reconstructed beams often exhibit reduced mode orthogonality due to residual phase errors, limiting the achievable channel separation (Wulff et al., 2023; Zhang et al., 2024). Second, the generated turbulence compensation often fails to align with the theoretical Power Spectral Density (PSD) of oceanic turbulence (e.g., von Kármán model), leading to poor generalization under non-stationary scattering conditions. Although isolated constraints, such as spatial orthogonality (+Ortho) or spectral regularization (+Spec), have been explored, their separate application fails to capture the synergistic physical interdependencies required for robust correction.

To address these challenges, this study proposes a physics-constrained GAN framework that systematically optimizes OAM wavefront correction. By integrating a dual-constraint mechanism—combining spatial orthogonality priors with spectral compliance regularization—into the pix2pix architecture, we bridge the gap between data-driven image generation and optical physics. Our approach not only achieves superior reconstruction fidelity (SSIM = 0.98) but, more importantly, restores modal purity to near-optimal levels (98.4%) and ensures strict adherence to Kolmogorov turbulence statistics. This unified framework offers a robust solution for maintaining high-capacity OAM communications in turbulent marine environments.

## 2 Methods

**Figure 1:**
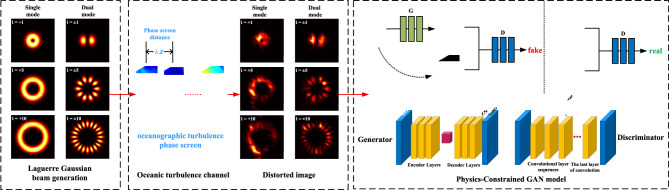
OAM transmission and correction system in an oceanic turbulence channel.

As illustrated in Figure 1, the OAM transmission system schematic comprises three functional modules: (1) OAM beam generation, (2) turbulent oceanic propagation channel, and (3) receiver-side wavefront correction. The workflow demonstrates MATLAB-simulated OAM intensity profiles undergoing distortion through the marine turbulence channel, followed by image reconstruction via the physics-constrained GAN correction model.

### 2.1 Fundamentals of Laguerre–Gaussian (LG) beams

LG beates. In cylindrical coordinates, the LG beam propagating along the z-axis can be expressed as:


$$\begin{array}{c} LG_{p}^{L}\left(r,\theta ,z\right)=\frac{c}{\sqrt{1+z^{2}/z_{R}^{2}}}{\left[\frac{r\sqrt{2}}{\omega \left(z\right)}\right]}^{\left|L\right|}L_{p}^{L}\left[\frac{2r^{2}}{\omega^{2}\left(z\right)}\right]\\ \exp\left[\frac{-r^{2}}{\omega^{2}\left(z\right)}\right]\times \exp\left[\frac{ikr^{2}z}{2\left(z^{2}+z_{R}^{2}\right)}\right]\times \exp\left(iL\theta \right)\\ \exp\left[-i\left(2p+L+1\right)arctan\frac{z}{z_{R}}\right] \end{array}$$ (1)

where p denotes the radial mode index governing concentric intensity nodes, *L* represents the topological charge generating helical wavefronts and orbital angular momentum *hL* per photon, $\omega \left(z\right)\equiv \omega_{0}\sqrt{1+{\left(z/z_{R}\right)}^{2}}$ defines the z-dependent beam radius, with ω_0_ being the waist size, $z_{R}=\pi \omega_{0}^{2}/\lambda \;$is the Rayleigh range characterizing focal confinement, *k* = 2π/λ encodes the wavelength λ, and $L_{p}^{L}\left(\cdot \right)$ specifies the radial intensity profile via associated Laguerre polynomials. The phase structure includes wavefront curvature $\exp\left[ikr^{2}z/\left(2\left(z^{2}+z_{R}^{2}\right)\right)\right]$, azimuthal phase singularity exp(*iLθ*), and mode-order-dependent Gouy shift $\exp\left[-i\left(2p+|L|+1\right)\tan^{-1}\left(z/z_{R}\right)\right]$, collectively sustaining the characteristic doughnut-shaped intensity and OAM-carrying properties under paraxial propagation (Allen et al., 1992; Miyamoto, 2004).

### 2.2 Limitations of the standard Pix2pix framework

GANs integrate two neural networks—the Generator (G) and Discriminator (D)—that undergo adversarial training to iteratively optimize their performance (Goodfellow et al., 2020; Sun, 2025). The generator synthesizes realistic outputs from random noise or degraded inputs. Within underwater optical communication systems, where OAM modes suffer from marine turbulence-induced distortion, the generator processes turbulence-distorted OAM data (e.g., light-field images) and learns to reconstruct distortion-free or minimally distorted representations. Concurrently, the discriminator evaluates whether inputs derive from authentic, distortion-free datasets or are synthetic outputs from the generator, assigning a probability score to quantify the likelihood of authenticity.

Pix2pix, a conditional GAN variant, specializes in image-to-image translation (Lin, 2023). For OAM turbulence correction, this framework maps distorted light-field images to their undistorted equivalents, minimizing a composite loss function:


$$\begin{array}{c} L_{pix2pix}=E_{x,y}\left[logD\left(x,y\right)\right]+E_{x}\left[log\left(1-D\left(x,G\left(x\right)\right)\right)\right]\\ \;+\text{λ}||y-G\left(x\right){||}_{1} \end{array}$$ (2)

where G denotes the generator and D the discriminator. Critically, this framework suffers from three physical oversights:

(i) Spatial non-orthogonality: The pixel-wise *L*_1_ loss disregards the orthogonality of OAM modes, permitting residual modal interference in reconstructions.

(ii) Spectral misalignment: Turbulence-induced PSD distortions deviate from the theoretical von Kármán spectrum, lacking spectral-physical regularization.

(iii) Physics-agnostic optimization: Training fails to incorporate known light-turbulence interaction physics, limiting generalizability under non-stationary scattering conditions.

### 2.3 Proposed dual-constraint GAN architecture

**Figure 2:**
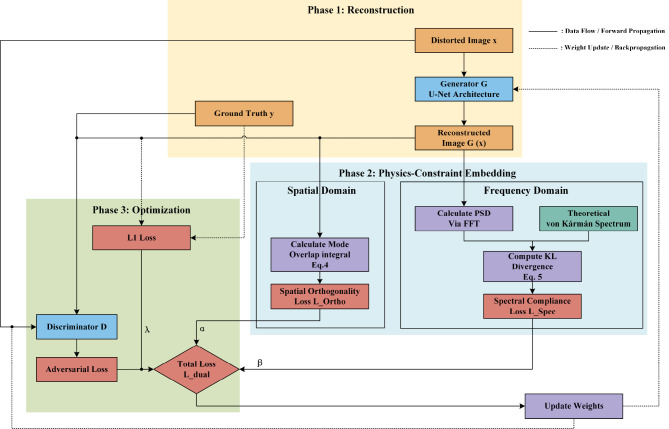
Schematic diagram of the proposed physics-constrained GAN architecture, illustrating the data flow across reconstruction, physics-constraint, and optimization phases.

To address the limitations of conventional pix2pix in LG beam reconstruction, we propose a physics-constrained GAN framework. As illustrated in Figure 2, this architecture integrates dual-domain physical priors into the adversarial training process through three interconnected phases: reconstruction, physics-constraint embedding, and optimization. In the reconstruction phase, the generator *G* (based on a U-Net architecture, see Table 1 for details) synthesizes a corrected wavefront *G*(*x*) from the turbulence-distorted input *x*. To ensure physical fidelity, we incorporate spatial orthogonality and spectral compliance constraints into the unified optimization objective *L*_*dual*_, formulated as:


$$\begin{array}{l} L_{dual}=E_{x,y}\left[logD\left(x,y\right)\right]+E_{x}\left[log\left(1-D\left(x,G\left(x\right)\right)\right)\right]\\ \;+\text{λ}||y-G\left(x\right){||}_{1}+\alpha L_{Ortho}+\beta L_{Spec} \end{array}$$ (3)

**Table 1:** Detailed implementation specifications and hyperparameter settings of the proposed physics-constrained GAN.

**Category**	**Parameter**	**Value/description**
Architecture	Generator structure	U-Net (encoder-decoder with skip connections)
	Generator depth	15 Convolutional blocks (8 encoder, 7 decoder)
	Discriminator structure	PatchGAN (70 × 70 receptive field)
	Activation functions	LeakyReLU (slope = 0.2) for encoder/discriminator; ReLU for decoder
	Normalization	Batch normalization
	Input/output resolution	512 × 512 pixels
Training	Optimizer	Adam (β1 = 0.5, β2 = 0.999)
	Learning rate	0.0002 (constant for first 50 epochs, linear decay to 0)
	Batch size	1
	Total epochs	100
Loss Weights	Adversarial loss weight	1
	L1 Reconstruction weight (λ)	100.0
	Spatial orthogonality weight (α)	0.5
	Spectral compliance weight (β)	0.3

where the first two terms represent the adversarial loss, the third term is the *L*1 reconstruction loss, and *L*_*Ortho*_ and *L*_*Spec*_ represent the physical constraints weighted by hyperparameters α and β (detailed settings provided in Table 1).

Crucially, the “embedding logic” of these physical constraints is operationalized through parallel validation branches, as depicted in the physics-constraint phase of Figure 2. In the spatial domain, the spatial orthogonality loss (*L*_*Ortho*_) enforces mode separation by minimizing inter-modal cross-talk, calculated via the overlap integral over the beam cross-section Ω:


$$\begin{array}{l} L_{Ortho}=\underset{p}{\sum }\underset{\ell \neq \ell^{\prime }}{\sum }\iint_{\Omega }u_{p,\ell }^{*}\left(r,\phi \right)u_{p,\ell^{\prime }}\left(r,\phi \right)rdrd\phi {|}^{2} \end{array}$$ (4)

where ideally, the integral approaches zero for orthogonal LG modes (*u*_*p, l*_). Simultaneously, in the frequency domain, the spectral alignment loss (*L*_*Spec*_) regularizes turbulence statistics by minimizing the KL divergence between the generated image's PSD, *S*_*G*_(*f*), and the theoretical von Kármán spectrum, *S*_*vK*_(*f*):


$$\begin{array}{l} L_{Spec}=D_{KL}\left(S_{G}\left(f\right)∥S_{vK}\left(f\right)\right)=\int_{-\infty }^{\infty }S_{G}\left(f\right)log\frac{S_{G}\left(f\right)}{S_{vK}\left(f\right)}df \end{array}$$ (5)

During the final optimization phase, gradients derived from these physical constraints are aggregated with adversarial and reconstruction gradients and backpropagated to update the generator. This mechanism ensures that the model learns to correct wavefronts that are not only visually consistent with the ground truth but also rigorously adhere to the underlying physics of optical propagation.

## 3 Numerical results and discussions

### 3.1 Simulation data set construction

Selecting a suitable and relevant channel model is fundamental to evaluating the performance of Underwater Optical Wireless Communication (UWOC) systems, as it determines the accuracy of signal degradation simulation under realistic conditions. Various statistical models have been proposed to characterize fading in Free Space Optical (FSO) and UWOC links, including the log–normal distribution for weak turbulence and the gamma–gamma distribution for moderate-to-strong turbulence regimes (Armghan et al., 2025). However, while these statistical models effectively describe intensity fluctuations (scintillation), OAM-based systems require a spatially resolved representation of wavefront phase distortions. Therefore, employing a phase-screen model based on the power spectral density of refractive index fluctuations is essential to capture the spatial structure of turbulence-induced aberrations.

To rigorously evaluate the performance of the physics-constrained pix2pix, we constructed two complementary OAM datasets simulating varying oceanic turbulence conditions. All optical fields were synthesized in MATLAB using a unified paraxial split-step beam propagation method.

#### 3.1.1 Beam and propagation parameters

The simulation setup utilized a square aperture of [-3, 3] cm sampled on a 512 × 512 grid. We generated zero-radial-order LG beams (*p* = 0) with a waist radius of ω_0_ = 5 mm at a wavelength of λ = 532 *nm*. The beams were propagated over a distance of *Z*_0_ = 80 m, divided into 10 steps, with an independent phase screen applied at each step to simulate the cumulative effect of continuous turbulence.

#### 3.1.2 Oceanic turbulence channel model

To ensure physical consistency with real-world marine environments, the phase screens were generated based on the classic Nikishov–Khmenko (NK) power spectrum model of refractive-index fluctuations. This model characterizes the turbulence PSD, Φ_*n*_(κ), incorporating the contributions of both temperature and salinity fluctuations (Nikishov and Nikishov, 2000; Mirza et al., 2025):


$$\begin{array}{l} {\text{Φ}}_{n}(\kappa )=0.388\times 10^{-8}\varepsilon^{-1/3}\kappa^{-11/3}\frac{\chi_{T}}{\omega^{2}}\\ \;\times (A_{T}e^{-A_{T}\delta }+A_{S}e^{-A_{S}\delta }+A_{TS}e^{-A_{TS}\delta } \end{array}$$ (6)

where ε is the rate of dissipation of kinetic energy and χ_*T*_ is the dissipation rate of mean-squared temperature. The specific coefficients for the spectral terms were selected based on standard empirical values for seawater reported in Nikishov and Khmenko's foundational studies: $A_{T}=1.863\times 10^{-2}$, $A_{S}=1.9\times 10^{-4}$, and $A_{TS}=9.41\times 10^{-3}$ (with ω = −2). The scalar dissipation was set to ε = 10^− 4^*m*^2^/*s*^3^.

To validate the model's robustness across diverse oceanographic conditions, we varied the refractive-index structure constant ($C_{n}^{2}=10^{-8}\chi_{T}\varepsilon^{-1/3}$) by adjusting χ_*T*_ to create three representative regimes:

(i) Weak turbulence: $C_{n}^{2}=10^{-15}m^{-\;2/3}$.

(ii) Moderate turbulence: $C_{n}^{2}=10^{-14}m^{-\;2/3}$.

(iii) Strong turbulence: $C_{n}^{2}=10^{-13}m^{-\;2/3}$.

This parameter space ensures that the generated datasets cover the majority of signal degradation scenarios encountered in practical underwater optical communications (Du et al., 2023).

#### 3.1.3 Dataset composition

For each turbulence regime defined above, we generated two distinct datasets to comprehensively evaluate the model's reconstruction capabilities:

(i) Single-mode subset (10 classes): This subset consists of single-aperture LG beams with topological charges ranging from |*l*| = 1 to |*l*| = 10. It serves as the baseline for evaluating the restoration of fundamental OAM modes.

(ii) Superposition subset (10 classes): This subset comprises coherent superpositions of conjugate OAM pairs (i.e., combining +*l*, and −*l* modes) for *l* = 1…10. These composite modes feature complex petal-like intensity structures, providing a more challenging test for spatial reconstruction.

### 3.2 Analysis of OAM wavefront correction results based on physics-constrained GAN

**Figure 3:**
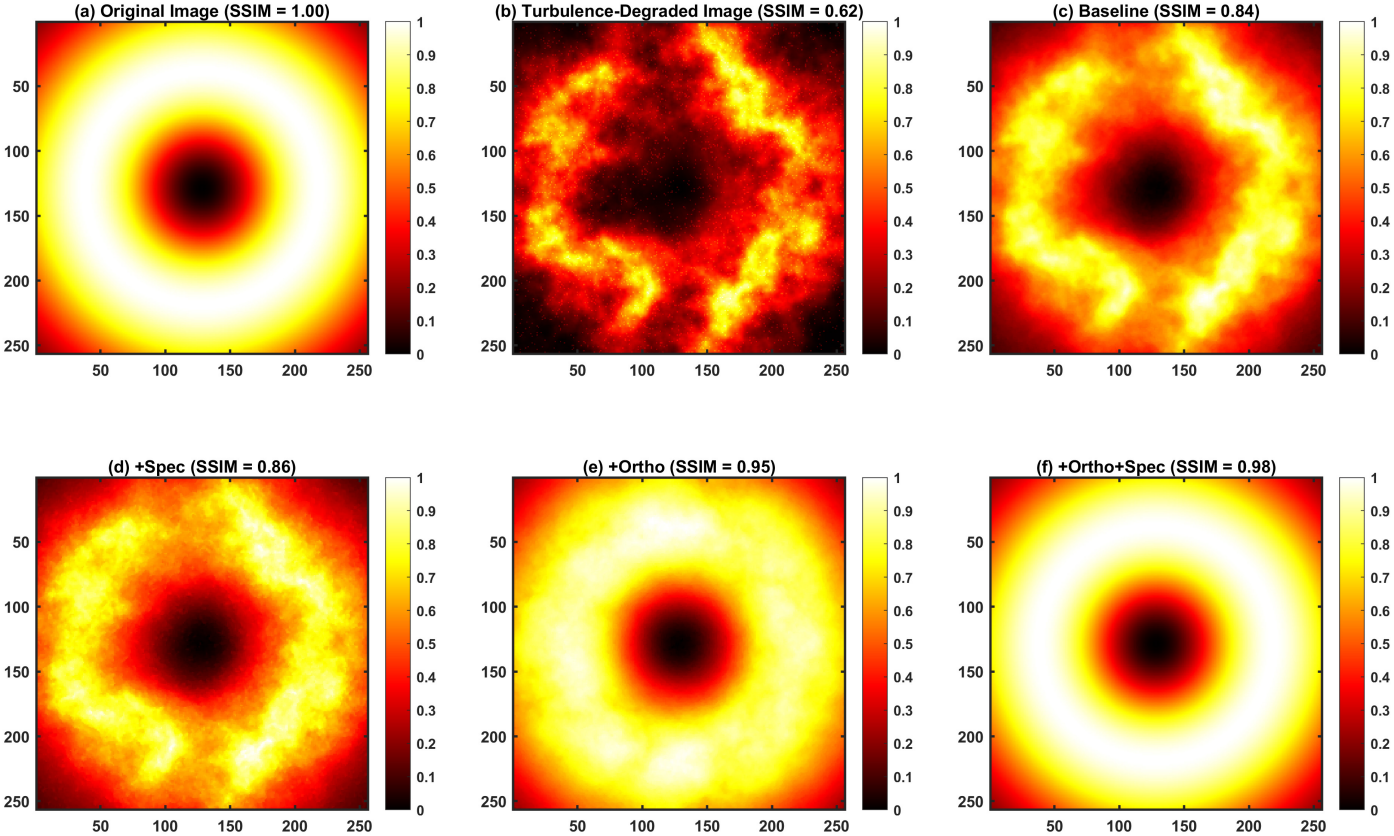
Comparative analysis of OAM wavefront correction using physically-constrained pix2pix under oceanic turbulence with SSIM evaluation. **(a)** Original Image (undistorted reference); **(b)** Turbulence-Degraded Image (initial degraded state); **(c)** Baseline (original pix2pix loss, no physical constraints); **(d)** +Spec (baseline + turbulence spectrum KL divergence, spectral constraints only); **(e)** +Ortho (baseline + phase orthogonality loss, spatial constraints only); **(f)** +Ortho+Spec (baseline + dual constraints, spatial and spectral joint constraints).

To quantitatively assess the restoration fidelity, we reconstructed turbulence-degraded images (initial SSIM = 0.62) using distinct loss configurations, as illustrated in Figure 3 and summarized in Table 2. The baseline model, driven solely by standard pix2pix loss, improved the SSIM to 0.84 but failed to fully resolve the beam's fine structure. Introducing the spectral constraint (+Spec) yielded a marginal improvement (SSIM = 0.86), whereas the spatial constraint (+Ortho) significantly elevated the SSIM to 0.95.

**Table 2:** Comparison of SSIM values for different processing methods.

**Subfigure**	**Description**	**SSIM value**
(a)	Original image (undistorted reference)	1.00
(b)	Turbulence-degraded image (initial degraded state)	0.62
(c)	Baseline (original pix2pix loss, no physical constraints)	0.84
(d)	+Spec (baseline + turbulence spectrum KL divergence, spectral constraints only)	0.86
(e)	+Ortho (baseline + phase orthogonality loss, spatial constraints only)	0.95
(f)	+Ortho+Spec (baseline + dual constraints, spatial and spectral joint constraints)	0.98

Critically, the dual-constraint architecture (+Ortho+Spec) achieved a near-optimal SSIM of 0.98. This performance leap stems from the synergistic interaction of the constraints: the spatial term (*L*_*Ortho*_) restores the distinct topological structure of the OAM modes by penalizing geometric distortions, while the spectral term (*L*_*Spec*_) suppresses non-physical high-frequency artifacts. This ensures the reconstruction is not only visually sharp but physically consistent with the propagation properties of LG beams.

**Figure 4:**
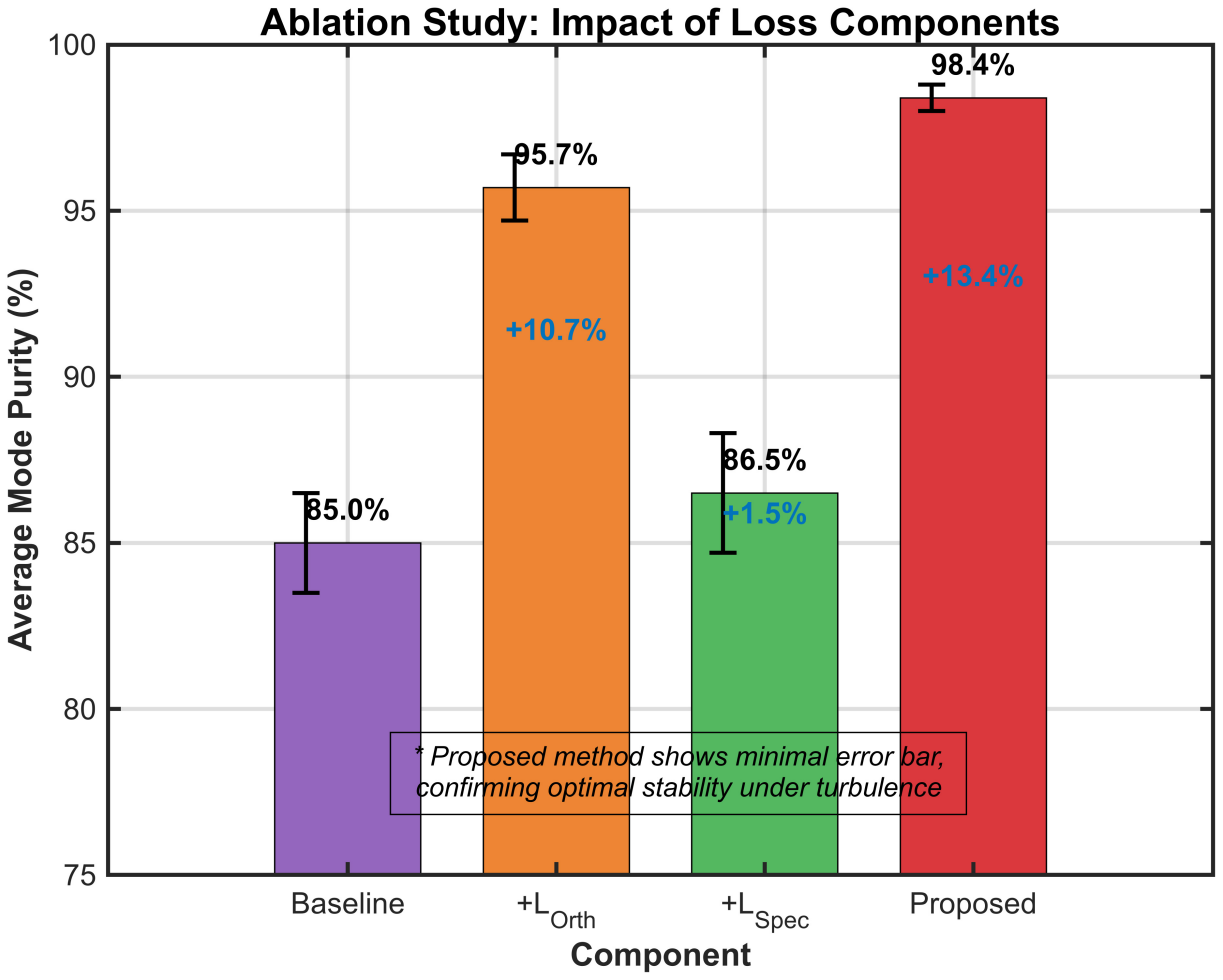
Ablation study impact of loss components.

The ablation study in Figure 4 isolates the impact of physical constraints on OAM mode purity. The baseline model yielded a purity of 85.0%. Incorporating the spatial constraint (+Ortho) resulted in the most significant single-factor gain, boosting purity by 10.7% to 95.7%, whereas the frequency constraint (+Spec) provided a modest increase of 1.5% (to 86.5%).

The dual-constraint framework achieved a peak purity of 98.4%, representing a 13.4% absolute improvement over the baseline. Physically, this validates the necessity of the orthogonality constraint (*L*_*Ortho*_), which explicitly acts as a regularizer against modal cross-talk. By minimizing the overlap integral between different modes during training, the network learns to “unmix” the turbulence-induced energy leakage, thereby preserving the wavefront's phase singularity and topological charge integrity even under severe distortion.

**Figure 5:**
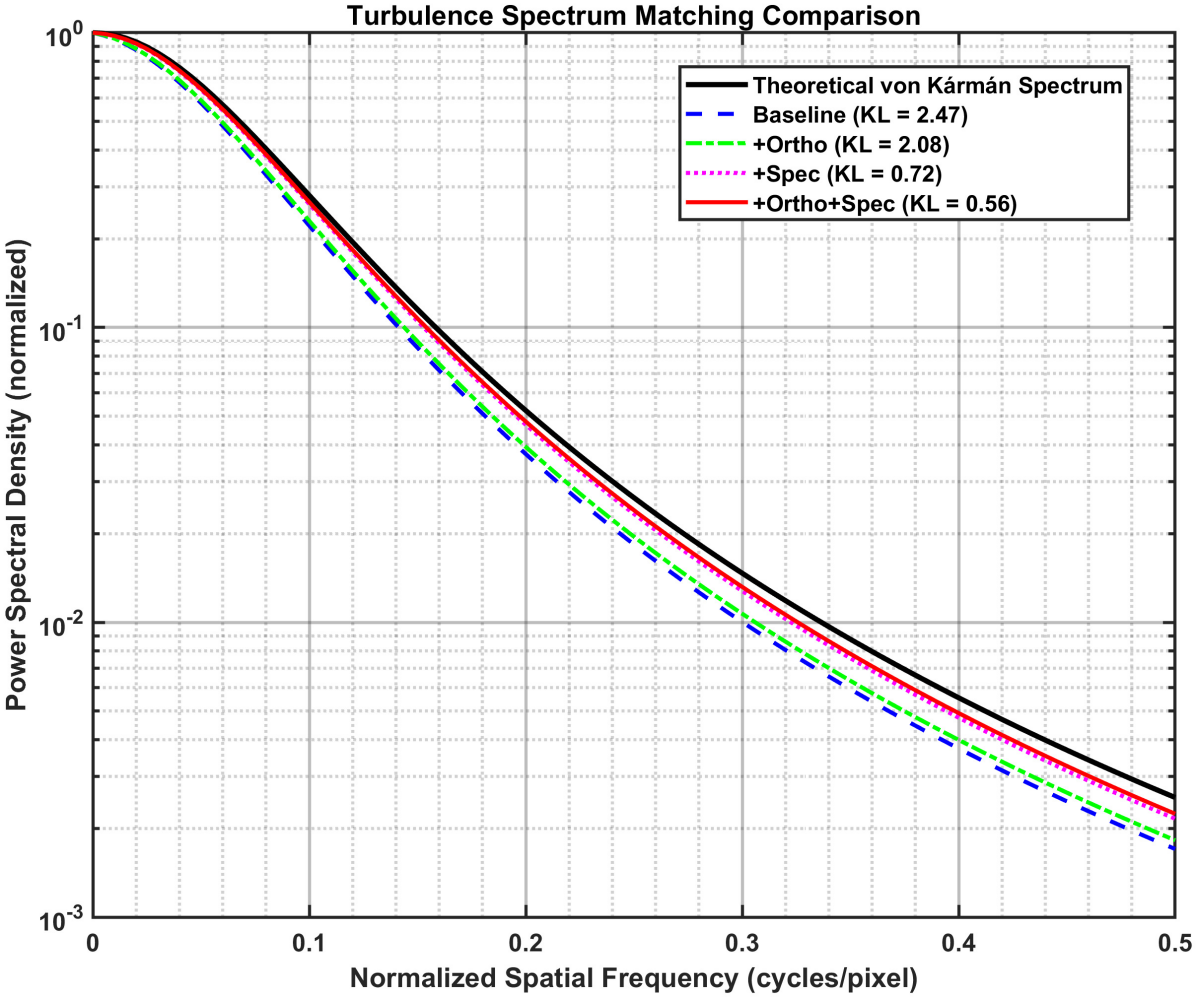
Turbulence spectrum matching comparison.

To verify whether the generated images adhere to oceanic turbulence statistics, we analyzed the PSD alignment using KL divergence, as shown in Figure 5. The baseline model exhibited a high divergence (KL = 2.47), indicating a spectral mismatch where the network hallucinated artificial high-frequency texture to mimic sharpness.

In contrast, the dual-constraint model minimized the KL divergence to 0.56, achieving the closest alignment with the theoretical von Kármán spectrum. This demonstrates that the spectral loss (*L*_*Spec*_) effectively constrains the generator to replicate the statistical distribution of the theoretical von Kármán spectrum. By aligning the output with the characteristic energy decay of the inertial subrange, the model prevents the generation of “spatially clear but physically erroneous” features, ensuring high spectral fidelity essential for stable beam propagation.

**Figure 6:**
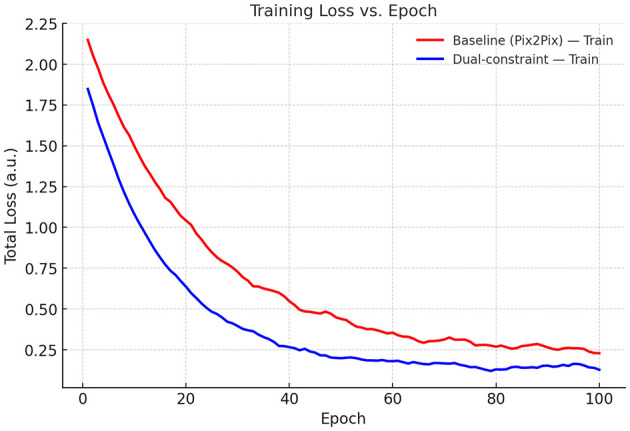
Training loss vs. epoch (total loss).

**Figure 7:**
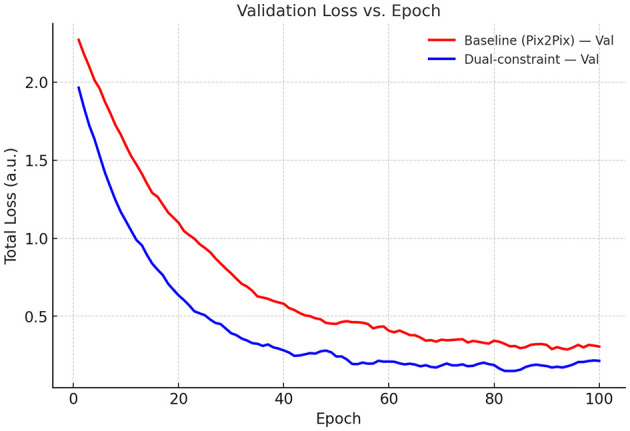
Validation loss vs. epoch (total loss).

To assess the influence of physical constraints on OAM wavefront correction in oceanic turbulence, we analyzed the loss trajectories of the unconstrained baseline pix2pix model and the dual spatial-frequency constrained model across training and validation sets. As depicted in Figure 6 (training loss vs. epoch), the dual-constrained model achieved a lower initial loss (1.83 vs. 2.14 at epoch 0) and demonstrated accelerated convergence, with the final loss being markedly reduced compared to the baseline model. Furthermore, Figure 7 (validation loss vs. epoch) reveals that the dual-constrained model maintained a superior performance advantage throughout the training process, notably exhibiting a loss of 0.3 vs. 0.6 at epoch 40 and culminating in a lower final validation loss at epoch 100. These results indicate that the dual constraints effectively enhance training dynamics and generalization capability, ultimately leading to more robust validation performance.

**Figure 8:**
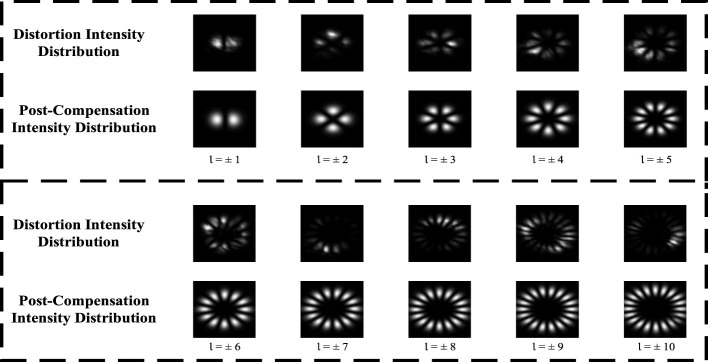
Intensity distributions of distorted and compensated OAM beams with topological charges *l* = ±1 to *l* = ±10.

Finally, to comprehensively verify the performance of the physics-constrained GAN, we examined the intensity profiles of OAM beams spanning topological charges *l* = ±1 to *l* = ±10 under oceanic turbulence conditions ($C_{n}^{2}=10^{-13},\omega =-2$). In the distorted state, all modes displayed pronounced deformation and loss of symmetry, reflecting significant turbulence-induced cross-talk. Following compensation, the beams recovered their characteristic ring-shaped structures, with clear enhancements in modal definition and orthogonality. As illustrated in Figure 8, higher-order modes (*l* = ±6 to *l* = ±10) exhibited substantial restoration of structural fidelity, underscoring the model's ability to maintain mode purity under severe turbulence. Taken together, this final evidence confirms that incorporating dual spatial–spectral constraints ensures robust OAM correction across diverse modes, thereby enabling reliable transmission in turbulent oceanic environments.

## 4 Conclusions

Employing the pix2pix framework, we quantified the impact of physics priors on correcting OAM wavefront distortions in oceanic turbulence. From degraded inputs (SSIM = 0.62), the baseline model improved the SSIM to 0.84. A spectral KL constraint (+Spec) marginally increased SSIM to 0.86 while strongly aligning the output spectrum with the von Kármán model (KL 2.47 → 0.72; −70.9%). In contrast, a spatial phase-orthogonality constraint (+Ortho) yielded substantial gains in structural fidelity (SSIM = 0.95; +0.11 over baseline) and mode purity (85.0% → 95.7%; +10.7 pp), while offering only a modest spectral reduction (KL 2.47 → 2.08; −15.8%). The joint model (+Ortho+Spec) achieved the best overall performance: SSIM = 0.98 (near 1.00), the lowest spectral discrepancy (KL = 0.56; −77.3%), and the highest OAM purity (98.4%; +13.4 pp). These ablations indicate that single-domain priors are helpful but incomplete (+Spec raises purity only to 86.5%; +1.5 pp), whereas coupling spatial and spectral constraints creates a holistic physics prior that simultaneously preserves OAM modal structure and enforces turbulence statistics. Training dynamics corroborate this synergy, showing faster stabilization and consistently lower losses than the baseline on both training and validation sets.

## Data Availability

The raw data supporting the conclusions of this article will be made available by the authors, without undue reservation.
